# HER2-positive metastatic breast cancer with brain metastases responds favorably to pyrotinib and trastuzumab-based treatment: A case report and literature review

**DOI:** 10.3389/fonc.2022.980635

**Published:** 2023-01-19

**Authors:** Min-long Chen, Wenjie Yu, Binbin Cui, Yijian Yu, Zhaosheng Ma

**Affiliations:** Department of Oncological Surgery, Taizhou Hospital, Wenzhou Medical University, Zhejiang, China

**Keywords:** breast cancer, brain metastase, pyrotinib, trastuzumab, treatment

## Abstract

For HER2-positive metastatic breast cancer patients with the brain involved at initial diagnosis, there was no standard regimen before 2022 when the HER2CLIMB trial published its final overall survival analysis, and the prognosis is relatively poor under the current treatment strategy. We herein reported a case of a female patient who was initially diagnosed with HER2-positive metastatic breast cancer with brain metastases, receiving pyrotinib and trastuzumab-based systematic therapy after palliative craniocerebral radiotherapy as the first-line systematic therapy. During the treatment, the tumor lesions showed obvious regression, and chemotherapy drugs were gradually removed from the regimen. The patient continued receiving trastuzumab and pyrotinib for HER2-targeted therapy. She had achieved more than 26 months of progression-free survival and the disease was stable during the evaluation in April 2022. Radiotherapy followed by dual HER2-targeted therapy of macromolecular monoclonal antibodies trastuzumab and micromolecular TKI pyrotinib plus chemotherapy could be an alternative option for this subtype of patients and need to be further verified by future clinical trials.

## Introduction

Breast cancer (BC) with overexpression of human epidermal growth factor receptor 2 (HER2) occurs in approximately 15% to 20% of all primary breast cancers, which were indicated to be more aggressive, easily metastasized, and to have poor prognosis (1). With the development of anti-HER2 systemic treatments, patients with HER2-positive breast cancer achieve long-term survival benefits, which also increase the incidence of brain metastases (BMs). BC with central nervous system (CNS) metastases has been reported in 15%–25% of BC patients, and BMs occur in 30%–55% of HER2-positive metastatic breast cancer (MBC) patients, which is much higher than other BC subtypes (2, 3). The median overall survival (OS) after the initial diagnosis of CNS metastases is poor, at 13.0 months (4).

Currently, locally directed therapy, such as surgical resection, stereotactic radiosurgery, and whole-brain radiation are initially considered for BMs. Dual-targeted therapy of pertuzumab and trastuzumab plus docetaxel or paclitaxelis is the first line recommendation for HER2-positive MBC by National Comprehensive Cancer Network (NCCN) guidelines (5). In the HER2CLIMB trial, tucatinib together with trastuzumab and capecitabine significantly improved OS and PFS in HER2-positive MBC, including those with BMs (6).

Pyrotinib is a novel oral pan-ErbB receptor tyrosine kinase inhibitor (TKI), which potently inhibits EGFR/HER1, HER2, and HER4 (7). It was approved for use in combination with capecitabine for the treatment of patients with HER2-positive metastatic BC in August 2018 in China. We herein reported a case of a female patient, who was initially diagnosed with HER2-positive metastatic BC with BMs, receiving pyrotinib and trastuzumab-based systematic therapy after palliative craniocerebral radiotherapy as the first-line systematic therapy. To the best of our knowledge, this is the first case reporting pyrotinib and trastuzumab-based systematic therapy as the first-line systematic therapy for HER2-positive metastatic BC with BMs.

## Case report

**Figure 1:**
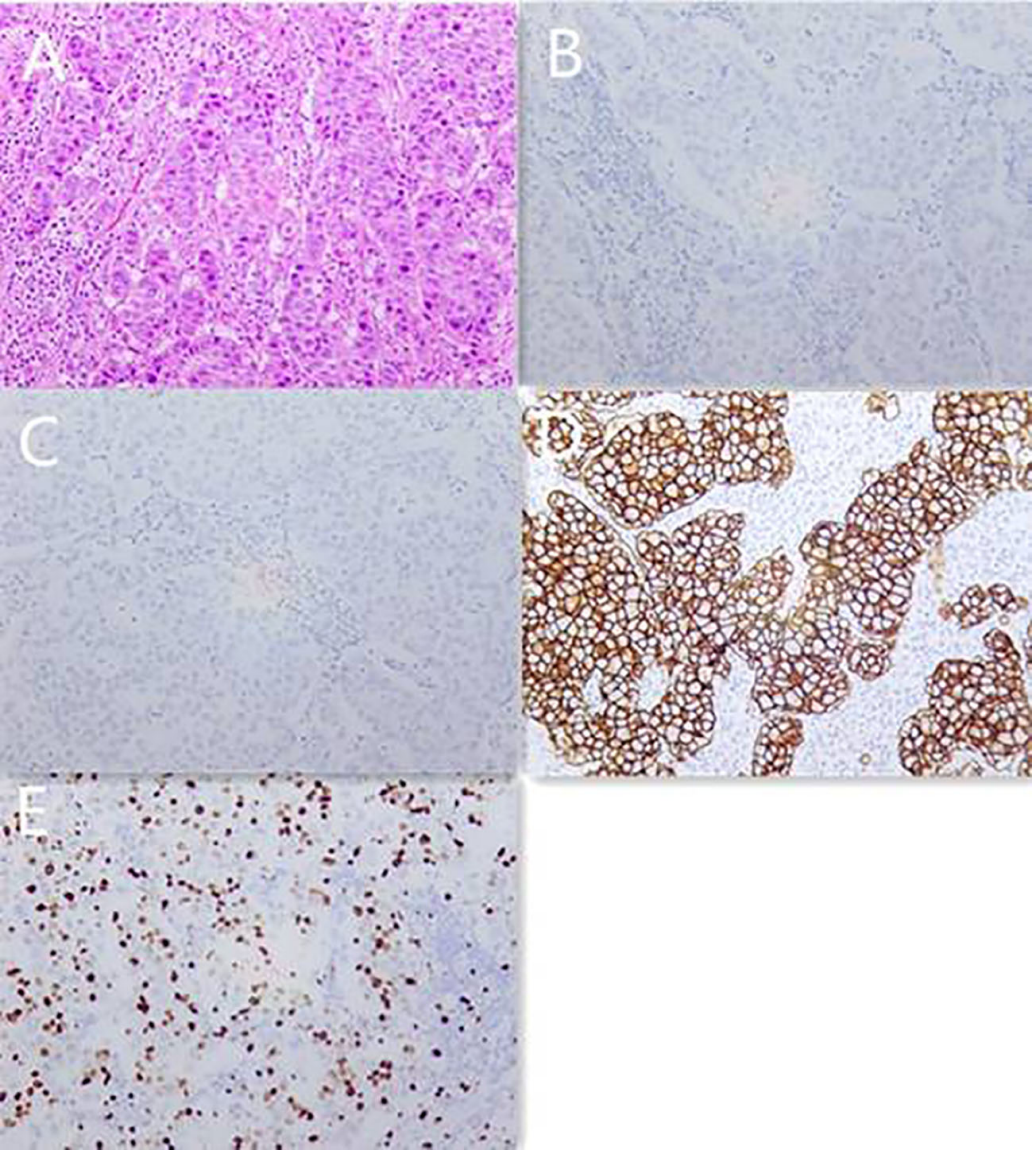
Pathology images and immunotherapy staining images of this patient in March 2020. **(A)** Pathology images of core needle biopsy. **(B)** ER immunotherapy staining image of core needle biopsy. **(C)** PR immunotherapy staining image of core needle biopsy. **(D)** HER2 immunotherapy staining image of core needle biopsy. **(E)** Ki-67 immunotherapy staining image of core needle biopsy.

**Figure 2:**
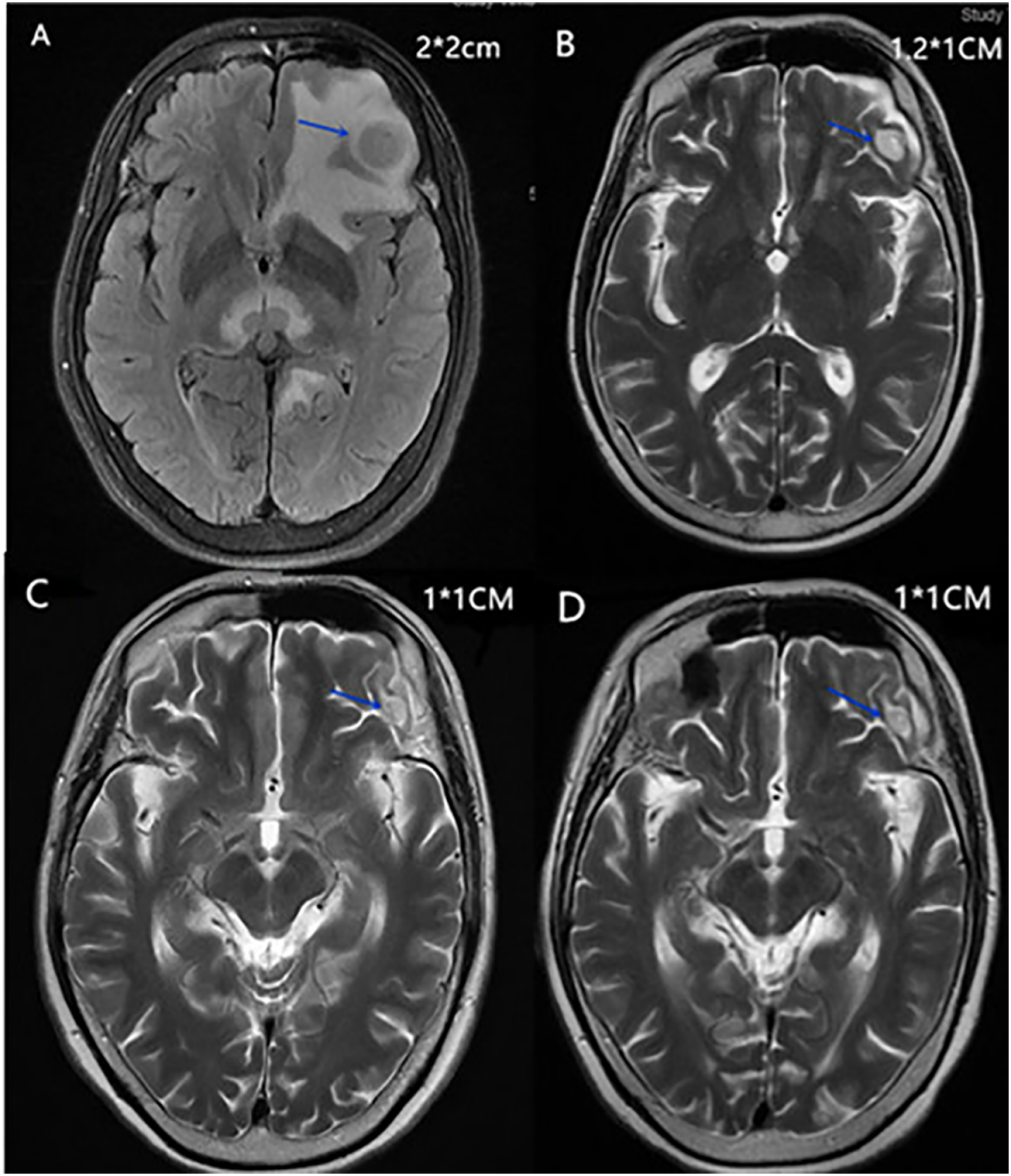
**(A)** Head MRI of the maximal mass in the left frontal lobe of the brain in April 2020. **(B)** Head MRI of the maximal mass in the left frontal lobe of the brain in May 2020. **(C)** Head MRI of the maximal mass in the left frontal lobe of the brain in August 2020. **(D)** Head MRI of the maximal mass in the left frontal lobe of the brain in April 2022.

**Figure 3:**
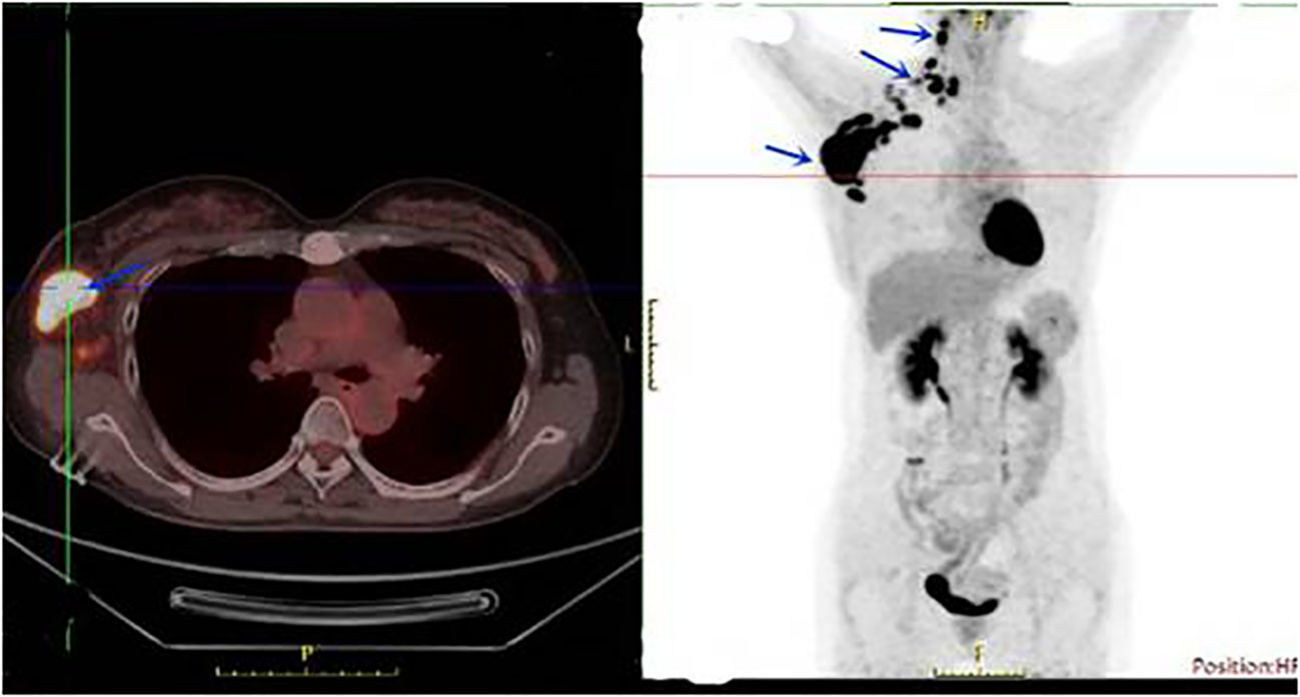
PET-CT showed multiple masses in the right axilla, right supraclavicular region, and spleen in March 2020.

A 56-year-old Chinese female patient presented to our outpatient department in March 2020, complaining of endurable headache and palpable nodules in the right axilla and supraclavicular region. A 5*6 cm hard tumor in the outer upper quadrant of the right breast and multiple enlarged lymph nodes in the right axilla and supraclavicular region were found in the physical examination. The pathological biopsy results of the maximal mass showed invasive ductal carcinoma (**Figure 1A**). Immunohistochemistry (IHC) analysis revealed ER (–), PR (-), HER2 (3+), GCDEF-15(+) (**Figures 1B-E**). Head magnetic resonance imaging (MRI) revealed multiple masses in brain parenchyma, the diameter of the maximal mass was 2 cm, located in the left frontal lobe of the brain (**Figure 2A**). Her serum cancer antigen 15-3 (CA15-3) was 126.1 U/ml (normal, <31 U/ml) while serum carcinoembryonic antigen (CEA) and serum cancer antigen 12-5 (CA12-5) were both in the normal level. Imaging assessment including chest computed tomography (CT) scan, and abdominal ultrasound indicated a negative result. PET-CT was then recommended and revealed multiple masses in the right axilla, right supraclavicular region, spleen, and brain (**Figure 3**). This patient was finally diagnosed with a metastatic breast cancer, T3N3M1, stage IV, HER2-positive.

This patient received palliative craniocerebral radiotherapy for 2 weeks in our hospital and the process was smooth. She then received systemic therapy of paclitaxel (175 mg/m^2^, d1, 1/21 d) and capecitabine (1000 mg/m^2^, bid, d1–d14/21 d) for chemotherapy plus trastuzumab (loading 8 mg/kg, then 6 mg/kg, d1, 1/21 d) and pyrotinib (400 mg, po, qd) for HER2-targeted therapy since April 2020. During the first course of systemic therapy, she had nausea, diarrhea, and loss of appetite, which could be recovered after symptomatic treatment, and chemotherapy-related hand-foot syndrome, which was endurable. Efficacy evaluation of the systemic therapy was performed at the beginning of the second course. Ultrasound revealed that all the enlarged lymph nodes in the axilla and supraclavicular region disappeared and the maximal mass in the breast was reduced to 2.1*0.8 cm. Head MRI revealed that the diameter of the maximal mass in the left frontal lobe of the brain was reduced to 1.2 cm (**Figure 2B**). CA15-3 also dropped back to normal level. Because of the excellent therapeutic effect, she continued the second course of previous systemic therapy.

During the second course of systemic therapy, the side effects of the drugs progressed. The patient complained most of insufferable loss in strength and acroanesthesia. At the beginning of the third course, ultrasound revealed a 1.8*1.0 cm mass in the breast and the mass in the left frontal lobe of the brain was similar with that of the last course in head MRI. CA15-3 was also in the normal level. Because of the severe side-effects of the drugs, capecitabine was first eliminated from the regimen since the third course. She continued another six courses of paclitaxel for chemotherapy plus trastuzumab and pyrotinib for HER2-targeted therapy. During this treatment, her breast mass showed continuous reduction and disappeared since the seventh course in ultrasound; the diameter of the maximal mass in the left frontal lobe of the brain reduced to 1.0 cm (**Figure 2C**) since the sixth course and the other small masses in the brain were completely relieved. Meanwhile, her strength quickly recovered, but she still felt the acroanesthesia even though symptomatic treatment was given. She stopped chemotherapy since the ninth course and continued trastuzumab and pyrotinib for HER2-targeted therapy. The latest treatment evaluation was carried out in April 2022, the mass in brain was stable, and no new lesion was discovered (**Figure 2D**). The patient was still receiving trastuzumab and pyrotinib for HER2-targeted therapy up to now. To now, the patient had received as long as 26 months of progression-free survival and the disease was still controlled well (**Table 1**).

**Table 1:** The brief course of treatment.

Date	Treatment	Maximal head mass (cm)	Mass in right axilla (cm)
2020.3	Diagnosed	2*2	5*6
2020.4	Palliative craniocerebral radiotherapy for 2 weeks	2*2	5*6
2020.4-	Two cycles of Paclitaxel, capecitabine plus trastuzumab and pyrotinib	2*2	5*6
2020.6-	Six cycles of paclitaxel, trastuzumab and pyrotinib	1.2*1	1.8*1
2020.11- 2022.4	Trastuzumab and pyrotinib	1*1	none

The maximal head mass was evaluated by MRI; the mass in right axilla was evaluated by ultrasonography.

## Discussion

Nowadays, patients with breast cancer brain metastasis are still lacking effective treatment and associated with poor outcomes, although substantial improvements have been achieved in the diagnosis and treatment (8, 9). Due to the existence of blood-brain barrier (BBB) and limited permeability, many chemotherapeutic drugs and macromolecular anti-HER2 targeted drugs exhibit restricted efficacy for intracranial lesions, such as paclitaxel, anthracyclines, trastuzumab, pertuzumab, and T-DM1 (10). Meanwhile, the good control of extracranial lesions by systemic treatment leads to long-term survival and also more BM occurrence. Thus, there is an urgent need for clinical strategies targeting BM for this type of breast cancer.

Radiotherapy is a common option for local control of brain lesions, reported to be able to change the permeability of the BBB (11). In the TBCRC 022 trial, neratinib plus capecitabine was considered to have synergistic effects with radiotherapy for HER2-positive breast cancer brain metastasis (12). In real world data, patients with BM could benefit in PFS and OS when receiving systemic therapy in combination with radiotherapy, compared with those not receiving radiotherapy (13, 14). In this case, the patient was recommended to receive palliative craniocerebral radiotherapy before systemic treatment.

Up to now, the anti-HER2 drugs are divided into three categories, including monoclonal antibodies, such as trastuzumab and pertuzumab; antibody-drug conjugates (ADC), such as T-DM1 and DS8201; and tyrosine kinase inhibitors (TKI), such as lapatinib, neratinib, tucatinib and pyrotinib. Trastuzumab-based therapy is still the standard regimen for the treatment of HER2-positive locally advanced or metastatic breast cancer recommended by the NCCN guidelines and Chinese Society of Clinical Oncology (CSCO) guidelines (5, 15). However, trastuzumab often shows primary or acquired resistance during or post treatment, and patients with BM were excluded from the criteria in the CLEOPATRA trial, which laid the foundation for dual-targeted therapy of pertuzumab and trastuzumab plus chemotherapy as the first-line treatment for HER2-positive metastatic breast cancer (16).

T-DM1 only contributed approximately 5.5 months of median PFS for patients with BM while it was confirmed to have an advantage for the PFS and OS for HER2-positive MBC in the EMILIA trial (17). Several studies reported that anti-HER2 monoclonal antibodies and HER2-directed antibody drug conjugates could improve survival in BC patients with BM, but considering the limited permeability into the BBB, their intracranial effects remain controversial (4, 16).

Compared with monoclonal antibodies, the physical features of small-molecule TKIs play an important role in allowing them to cross the BBB, thereby improving drug concentrations in the brain, indicating that TKIs could be a rational therapeutic approach to treat CNS metastases (18, 19). Tucatinib, a small-molecule oral tyrosine kinase inhibitor (TKI) that is highly selective for HER2, was approved by the FDA in April 2020 for use in patients who have received one or more prior anti-HER2-based regimens in the metastatic setting. In the HER2CLIMB study, compared with placebo, the addition of tucatinib to trastuzumab and capecitabine reduced the risk of intracranial progression by 68% (hazard ratio [HR], 0.32; 95% CI, 0.22–0.48; p = .0001), and reduced the risk of death by 42% (OS HR, 0.58; 95% CI, 0.40–0.85; p = .005) among BMs group, providing a clinically meaningful survival benefit (6, 20). Unfortunately, tucatinib was not available in China in 2020 and not even nowadays.

Pyrotinib is another novel micromolecular oral pan-ErbB receptor TKI, inhibiting HER1, HER2, and HER4 (7), which was first approved in China for use in combination with capecitabine for the treatment of patients with HER2-positive MBC who had previously received anthracycline or taxane chemotherapy. An open-label phase II study organized in China demonstrated that pyrotinib plus capecitabine had a significantly longer PFS (18.1 *vs*. 7.0 months, p < 0.001) and higher objective response rate (ORR) (78.5% *vs*. 57.1%, p < 0.05) than lapatinib plus capecitabine in MBC patients (21). The PHOEBE study conducted a similar result for those patients who had been previously treated with trastuzumab and taxane and/or anthracycline (22). Furthermore, neratinib plus capetabine achieved 8.8 months of median PFS as a third or later line therapy, and neratinib plus paclitaxel achieved 12.9 months of median PFS as a first-line treatment, suggesting the potentially comparable efficacy of pyrotinib to neratinib (23, 24). Among patients with brain metastases, pyrotinib was reported to have a better PFS benefit than monoclonal antibodies in real-world study (25, 26).

For this patient, there is no standard regimen, considering the heavy tumor burden and the metastases in the brain; dual HER2-targeted therapy plus chemotherapy was preferentially recommended in the purpose to rapidly contract the tumor. In the 2020 CSCO guidelines, the regimen of TXH (taxel, capecitabin, trastuzumab) or THP (taxel, trastuzumab, pertuzumab) was first recommended for those HER2-positive MBC patients without trastuzumab pretreated. Furthermore, the unavailability of TDM-1 and neratinib in China at that time and the high price of Pertuzumab prevent the patient from considering the clinical application of these drugs. Pyrotinib was finally added in the regimen of TXH. During the treatment, the tumor showed obvious regression, especially the extracranial lesions, which disappeared after six cycles of treatment. Chemotherapy drugs were gradually removed from the regimen because of the progressed side effects. Till now, the patient is still receiving trastuzumab and pyrotinib for HER2-targeted therapy and the disease is stable during evaluation.

## Conclusion

For HER2-positive MBC patients with brain involved at initial diagnosis, the current treatment strategy results in relatively poor prognosis. Radiotherapy followed by dual HER2-targeted therapy of macromolecular monoclonal antibodies trastuzumab and micromolecular TKI pyrotinib plus chemotherapy could be an alternative option for this subtype of patients and needs to be further verified by future clinical trials.

## Data availability statement

The original contributions presented in the study are included in the article/supplementary material. Further inquiries can be directed to the corresponding author.

## Author contributions

MC, WY, BC and ZM acquired the data and prepared the manuscript. YY performed histological examinations.MC and ZM performed data analysis and interpretation. All authors contributed to the article and approved the submitted version.
